# Magnitude of unmet need for family planning and associated factors among women in the extended postpartum period in Dabat district, Northwest Ethiopia. evidence from Dabat demographic health surveys

**DOI:** 10.1186/s12889-023-16046-3

**Published:** 2023-06-12

**Authors:** Abebaw Addis Gelagay, Wubshet Debebe Negash, Tadele Biresaw Belachew, Desalegn Anmut Bitew, Elsa Awoke Fentie, Abebaw Gebeyehu Worku, Debrework Tesgera Bashah, Nigusie Birhan Tebeje, Mignote Hailu Gebrie, Hedija Yenus Yeshita, Endeshaw Adimasu Cherkose, Birhanu Abera Ayana, Ayenew Molla Lakew, Desale Bihonegn Asmamaw

**Affiliations:** 1 Department of Reproductive Health, Institute of Public Health, College of Medicine and Health Sciences, University of Gondar, Gondar, P.O.Box: 196, Ethiopia; 2 Department of Health Systems and Policy, Institute of Public Health, College of Medicine and Health Sciences, University of Gondar, Gondar, Ethiopia; 3 School of Nursing, College of Medicine and Health Sciences, University of Gondar, Gondar, Ethiopia; 4 School of Midwifery, College of Medicine and Health Sciences, University of Gondar, Gondar, Ethiopia; 5 Department of Obstetrics and Gynecology, Zewuditu Memorial Hospital, Addis Ababa, Ethiopia; 6 Department of Epidemiology and Biostatistics, Institute of Public Health, College of Medicine and Health Sciences, University of Gondar, Gondar, Ethiopia

**Keywords:** Unmet need, Family planning, Postpartum women, Dabat, Ethiopia

## Abstract

**Background:**

Due to additional roles and emotional changes that occur during postpartum period, women use contraceptives differs from other times in their life. However, there is limited information about the unmet need for family planning (FP) among women in the extended postpartum period in the study area. Therefore, this study aimed to assess magnitude of unmet need for family planning and associated factors among women in the extended postpartum period in Dabat district, Northwest Ethiopia.

**Methods:**

A secondary data analysis was performed using the Dabat Demographic and Health Survey 2021. A total sample of 634 women during the extended postpartum period was included in this study. Stata version 14 statistical software was used for data analysis. Descriptive statistics were described using frequencies, percentages, mean, and standard deviation. Multicollinearity was tested using the variance inflation factor (VIF) and we computed Hosmer and Lemeshow goodness of fit. Both bivariable and multivariable logistic regression analyses were carried out to determine the association between independent variables and outcome variable. Statistical significance was declared at a p-value ≤ 0.05 with a corresponding 95% confidence interval.

**Results:**

The overall unmet need for FP during the extended postpartum women was 42.43% (95% CI: 38.62, 46.33), of which 33.44% was unmet need for spacing. Place of residence (AOR = 2.63, 95%CI: 1.61, 4.33), place of delivery (AOR = 2.09, 95%CI: 1.35, 3.24), and availability of radio and or TV (AOR = 1.58, 95% CI: 1.22, 2.13) were significantly associated with unmet need for family planning.

**Conclusion:**

The magnitude of unmet need for family planning among women during the extended postpartum period in the study area was high when compared to the national average and the United Nations sphere standard of unmet need for family planning. Place of residence, place of delivery, and availability of radio and or TV were significantly associated with unmet need for family planning. Hence, the concerned bodies are recommended to promote intuitional delivery and give spatial attention to those who are residing in rural areas and to those who have had no media exposure in order to reduce the unmet need for family planning among postpartum women.

## Introduction

Extended postpartum period refers to the period between birth and one year after delivery, during which the reduction of high-risk pregnancy can prevent child and maternal mortality and morbidity [1]. The World Health Organization (WHO) defines unmet family planning (FP) needs as those who are fertile and sexually active but are not using contraception, reporting not wanting any more children or wanting to delay having more children [2]. Among the indicators used to measure universal reproductive health coverage, it is a vital one [3]. Among the many aspects of women’s rights is family planning, which is key to their reproductive health [4].

There are approximately 214 million women worldwide who have unmet family planning [5]. More than 200 million women in developing countries do not have access to family planning [6]. More than one in five married women in Ethiopia have unmet needs for family planning, according to the 2016 Ethiopia Demographic and Health Survey (EDHS). With a total fertility rate of 4.6 [7]. Studies conducted in different regions of Ethiopia have also revealed that the unmet need for family planning ranged from 16.2 to 44% [8–11].

Unmet need for FP has been linked to high fertility, unintended pregnancies, and unsafe abortions, according to previous research [12]. Globally, there are about 80 million unintended pregnancies per year. As a result, mothers, children, and society as a whole suffer [13]. For instance, each year, an estimated 18 million unsafe abortions occur in low- and middle-income countries. [14]. It is still common for women in low- and middle-income countries to have unplanned pregnancies, despite several interventions [15, 16]. The lack of family planning also leads to a short birth interval that increases the morbidity and mortality risk for both the newborn and the preceding child [17].

The unmet need for family planning is influenced by factors such as the resumption of menses, confusion when fertility returns, the unpredictability of the timing of the onset of intercourse, not discussing family planning methods with partners, and a lack of knowledge of family planning. Moreover, place of residence, respondent’s education, respondent’s work status, being visited by a health worker, parity, and place of delivery were also other factors associated with unmet need for FP [8–10, 18, 19].

Although extensive research has been carried out on the unmet need for family planning, most of these studies were limited to reproductive-age women and focused on specific areas [20–23]. Even if some studies are conducted exclusively on postpartum women [24–27], almost all of these studies are done in urban areas and highly accessible service areas. The current study used Dabat demographic health survey data and was done only among postpartum women, which included both rural and urban areas and covered a wide area, which differed from the previous studies. Moreover, the current study tried to assess additional factors such as receiving family planning counselling during pregnancy and attending postnatal care. There is no study conducted in our study area on the utilization of postpartum family planning and associated factors. Therefore, the focus of this study is unique and has aimed to address two questions about the unmet need for family planning in the Dabat district: (a) investigate the unmet need for family planning among postpartum women; and (b) identify associated factors that lead women not to utilize family planning in the study areas. This study contributes to the field of family planning by identifying different factors of unmet need for family planning. The finding is also important for policymakers and program planners to realize women’s need for family planning in the study area particularly for postpartum women.

## Materials and methods

### Study setting

The study was conducted in Dabat, Dabat is located in northwest Ethiopia, which is about 76 km away from Gondar (Zonal town) to the north. According to the Dabat district health office report, the projected estimate of the population in the district was 189,944 in 2020/2021. There were a total of 44,789 reproductive women, 25,718 under-five children, and 6,401 infants. The Dabat district has a total of 36 *kebeles* (the smallest administrative units in Ethiopia), of which 31 are rural *kebeles*. In the district, there are 6 health centers and 29 health posts. The Dabat Demographic Health survey (DDHS) is one of the six health and Demographic Surveillance Systems in Ethiopia. The DDHS consists of 13 *kebeles* (9 rural and 4 urban). The DDHS sites are determined by University of Gondar, Institute of Public Health from Dabat district representing each agro-ecological zones of the district.

### Study design and period

This study used a secondary data of DDHS conducted from December 10, 2020, to January 10, 2021.

### Sampling technique and sample size determination

This study applied a survey method to identify and select eligible study participants. A total sample of 634 women during the extended postpartum period were included in the study.The study population was all mothers in Dabat demographic health survey sites who gave birth within one year before the survey and who were available in their homes during the survey. Women who gave stillbirths within the last year and those who gave live birth but were not alive during the survey were excluded from this study.

### Study variables

#### Dependent variable

The dependent variable for this study was unmet need for family planning during extended postpartum period, which was generated from constructed Dabat demographic health survey variables. It is the sum of unmet need for limiting and spacing. Women who have unmet needs if they want to delay or limit future pregnancy but do not use any form of family planning. The dependent variable was a binary variable. Those with an unmet need for spacing or limiting were coded as 1, while those using FP methods for spacing and or limiting were coded as 0 [26, 28].

#### The independent variables

In this study, we consider different independent variables such as Age of the mothers, residence, occupation of the mothers, education of the mothers, wealth index, availability of radio and or TV, husband’s education, husband occupation, parity, ANC visit, place of delivery, attending at least one PNC, fertility desire, have partner, and receiving family planning counselling during ANC visit were included.

### Data processing and analyses

Stata version 14 statistical software was used for data analysis. Descriptive statistics were described using frequencies, percentages, mean, and standard deviation, which were further presented using tables, figures, and text. Normality tests such as kurtosis and skewness were employed to see the normal distribution of the variables and to identify which summary measures were appropriate to use. Multicollinearity was tested using the variance inflation factor (VIF), and we got a VIF of less than five for each independent variable with a mean VIF of 1.69, indicating there was no significant multicollinearity between independent variables [29]. A binary logistic regression analysis was carried out to identify factors associated with unmet need for FP. Those variables with P-value ≤ 0.25 from the bivariable analysis were entered into a multivariable logistic regression model. Before performing multivariable logistic regression, we computed Hosmer and Lemeshow goodness of fit, and the model was adequate with a p-value of 0.98. Odds ratio with 95% confidence intervals was computed to see if there was an association between unmet need for family planning and associated factors. A P-value of 0.05 was used to declare statistical association.

## Results

### Sociodemographic characteristics

A total of 634 women during in the extended postpartum period were included. The mean age of the study participants was 29.55 with (± 5.40) years old. More than half (53.94%) of the study participants were under the age group of 25–34 years, 341 (53.79%) of women had no formal education. Moreover, 409 (64.51%) of women were rural dwellers and 150 (23.66%) of women fell in the poor wealth categories (Table 1).

**Table 1:** Sociodemographic characteristics of respondents in Dabat, District, Northwest Ethiopia

Variables	Frequency (n)	Percentage (%)
Age of the mother		
15–24	125	19.72
25–34	342	53.94
35–49	167	26.34
Educational status of the mother		
No formal education	341	53.79
Primary education	148	23.34
Secondary education	145	22.87
Occupation of the mother		
Housewife	545	85.96
Government employ	42	6.62
Farmer	17	2.68
Other	30	4.73
Education of the husband		
No formal education	324	51.10
Primary education	177	27.92
Secondary education and above	133	20.98
Occupation of the husband		
Farmer	469	73.97
Government employee	102	16.09
Daily labor	63	9.94
Place of residence		
Rural	409	64.51
Urban	225	35.49
Wealth index		
Poorest	93	14.67
Poorer	150	23.66
Middle	112	17.67
Richer	129	20.35
Richest	150	23.66
Availability of radio and or TV		
Yes	150	23.66
No	484	76.34

### Obstetric and reproductive factors

About 64.35% and 58.83% of the mothers were multipara and had an ANC visit for their index child, respectively. More than half (54.89%) of the participants gave birth at the health institutions (Table 2).

**Table 2:** Obstetric and reproductive factors of respondents in Dabat District, Northwest Ethiopia

Variables	Frequency (n)	Percentage (%)
Parity		
Primipara	99	15.62
Multipara	408	64.35
Grand multipara	127	20.03
ANC visits		
Yes	373	58.83
No	261	41.17
Place of delivery		
Home	286	45.11
Health institution	348	54.89
Attending at least one PNC		
Yes	210	33.12
No	424	66.88
Receiving family planning counseling during pregnancy		
Yes	80	12.62
No	554	87.38
Fertility desire		
Yes	508	80.13
No	126	19.87
Have partner		
Yes	617	97.32
No	17	2.68

### Unmet need for family planning

**Fig. 1:**
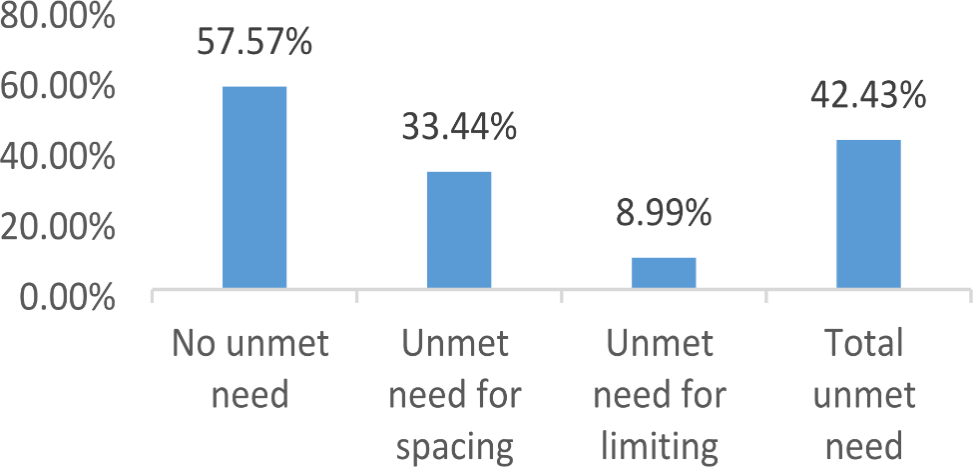
Unmet need for FP among women during in the extended postpartum period in northwest in Ethiopia **List of tables**

In this study, the overall unmet need for FP among extended postpartum mothers was 42.43% (95% CI: 38.62, 46.33), of which 33.44% was unmet need for spacing (Fig. 1).

### Factors associated with unmet need for family planning

In multivariable logistic regression, place of residence, place of delivery, and availability of radio and or TV were statistically significant factors for unmet need for family planning. Accordingly, unmet need for family planning was 2.63 times higher among women during in the postpartum period residing in rural areas (AOR = 2.63, 95%CI: 1.61, 4.33) compared to women during in the postpartum period residing in urban areas.

Similarly, the odds of postpartum mothers having unmet need for family planning was 2.09 (AOR = 2.09, 95%CI: 1.35, 3.24) times higher among postpartum mothers who gave birth at home compared to postpartum mother who gave birth at health institutions.

The likelihood of unmet need for family planning was 1.58 times higher (AOR = 1.58, 95% CI: 1.22, 2.13) among respondents who had radio and or TV in their home as compared to their counterparts (Table 3).

**Table 3:** Association of unmet need for family planning with different characteristics of women during in the postpartum period in Dabat, District, Northwest Ethiopia

Variables	Unmet need for FP		COR (95%CI)	AOR (95%CI)
	Yes	No		
Age of the mothers				
15–24	55	70	1	1
25–34	138	204	0.86 (0.57, 1.30)	0.99 (0.63, 1.56)
35–49	76	91	1.06 (0.67, 1.69)	1.09 (0.63, 1.87)
Residence				
Rural	215	194	3.50 (2.44, 5.04)	2.63 (1.61, 4.33)
Urban	54	171	1	1
Education of the mother				
No formal education	159	182	1.88 (1.25, 2.83)	0.86 (0.48, 1.54)
Primary education	64	64	1.64 (1.02, 2.64)	0.93 (0.52, 1.65)
Secondary education and above	46	99	1	1
Have partner				
Yes	258	359	1	1
No	11	6	2.55 (0.93, 6.98)	3.40 (0.97, 8.85)
Wealth index				
Poor	123	120	1.78 (1.25, 2.52)	1.04 (0.66 (1.64)
Middle	44	68	1.12 (0.72, 1.76)	0.91 (0.53, 1.54)
Rich	102	177	1	1
Availability of radio and or television				
Yes	46	104	1	1
No	223	261	1.93 (1.31, 2.85)	1.58(1.22, 2.13)
ANC visits				
Yes	151	222	1	1
No	118	143	1.21 (0.88, 1.67)	0.86 (0.57, 1.27)
Place of delivery				
Health institution	111	237	1	1
Home	158	128	2.64 (1.91, 3.65)	2.09 (1.35, 3.24)
Family planning counseling during pregnancy				
Yes	29	51	1	1
No	240	314	1.34 (0.83, 2.18)	0.98 (0.56, 1.71)
Attending at least one PNC				
Yes	92	118	1	1
No	177	247	0.92 (0.66, 1.28)	0.80 (0.53, 1.21)
Fertility desire				
Yes	212	296	1	1
No	57	69	1.15 (0.78, 1.71)	1.17 (0.75, 1.81)

*Significant at p < 0.05, **Significant at p < 0.01, FP = family planning, PNC = postnatal care and ANC = antenatal care, Hosmer and Lemeshow goodness of fit (p-value = 0.98)

## Discussion

This study was conducted to examine the magnitude and associated factors of the unmet need for family planning among women during in the extended postpartum period in Dabat district, Northwest, Ethiopia. In the current study, the unmet need for FP was found to be 42.43%. This finding is in line with study done in Dessie, Ethiopia 44% [11]. However, the current study is higher than the national report, which was 22% [7]. And higher than previous studies done in Ethiopia [9, 30, 31]. It is higher from the national target of reducing the level of unmet need for FP to 10% by 2020 [32]. The magnitude was higher compared with the United Nations sphere standard of unmet need for FP, which is considered high if greater than 25%, and the global estimate of unmet need for FP among reproductive age women 24.3% [33, 34]. The finding was also higher than studies conducted in Zambia 21% [35], and Bangladesh 13.50% [36]. The reason for the high unmet need for FP in this study compared to other studies might be the difference in health service coverage. In the current study, most of the places are rugged (hard to reach area), making it difficult to distribute the service easily, and there is a lack of infrastructure due to the geographic areas. In addition, it may be due to the deference in the sociodemographic factors of the study participants. For example, the difference in educational status, only 10.4% and 19.3% women in the Zambia [35] and Bangladesh [36] studies had no formal education, respectively. Previous studies done in Ethiopia 27 − 41.6% [9, 31]. Compared to 53.94% in this study. Education can increase women’s awareness and level of understanding about the risk of being pregnant in the postpartum period. The other reason for the higher unmet need for family planning in this study might be the difference in availability of media in their home. In the current study, only 45.11% of women have mass media (radio and or TV) in their home, but more than 57% of women who participated in the above studies have mass media (radio and or TV) in their home.

The odds of having an unmet need for FP among women during in the extended postpartum period living in rural areas were 2.63 times higher than their counterparts. This is similar to studies conducted in Ethiopia [37], Nigeria [38], and Bangladesh [39]. This might be due to a variety of reasons; in Ethiopia; rural residents have poor health service accessibility and low awareness of contraceptives due to the fact that rural women are less educated, have limited access to mass media, have insufficient income, and poor infrastructure, which has a negative impact on family planning use [7, 40]. Moreover, evidence revealed a high concentration of sexual and reproductive health services delivery in an urban area in Ethiopia [30, 33, 41].

The current study also revealed that place of delivery is negatively associated with unmet need for FP. The odds of unmet need for FP was higher among women who gave birth at home compared to those who gave birth at health institutions. This could be due to women who gave birth at the home are less likely to be counseled about postpartum FP and initiated to use a FP [42].

In this study, availability of Radio and/or TV in the participant home was found to be one of the associated factor with unmet need for FP. Women who had no media (TV and/or Radio) in their homes were more likely to have unmet need for FP compared to those women who had Radio and/or TV in their home. This finding is in line with Southwest Ethiopia [30], further DHS analysis [43], Mali [44], and Nigeria [45]. The possible justification might be that women with no media exposure might not have a better understanding of contraception, which cannot have a positive change in their attitude toward contraception [45, 46]. The study indicates that media exposure will reduce the barriers to access and use of health care services, including contraception.

Social desirability bias and recall bias might be introduced. Because, those post-partum women were asked about their recent pregnancy. Therefore, they might not exactly remember or sometimes hide the facts. To minimize these, vital events in women’s life were asked, and female data collectors were recruited. The current study was conducted on women only, but it would be better to include male partners, health care providers, and institutional delivery services to identify factors affecting unmet need for FP. Moreover, since the survey is cross-sectional study design, causality cannot be established for the findings.

## Conclusion

The magnitude of unmet need for family planning among women during the extended postpartum period in the study area was high when compared to the national average and the United Nations sphere standard of unmet need for family planning. Place of residence, place of delivery, and availability of radio and or TV were significantly associated with unmet need for family planning. Hence, the concerned bodies are recommended to promote intuitional delivery and give spatial attention to those who are residing in rural areas and to those who have had no media exposure in order to reduce the unmet need for family planning among postpartum women.

## Data Availability

All the data generated in this study are included in this manuscript. The datasets used and analyzed to produce the current manuscript will be obtained from the corresponding author upon request.
